# Heparan Sulfate Deficiency in Cartilage: Enhanced BMP-Sensitivity, Proteoglycan Production and an Anti-Apoptotic Expression Signature after Loading

**DOI:** 10.3390/ijms22073726

**Published:** 2021-04-02

**Authors:** Matthias Gerstner, Ann-Christine Severmann, Safak Chasan, Andrea Vortkamp, Wiltrud Richter

**Affiliations:** 1 Research Centre for Experimental Orthopaedics, Orthopaedic University Hospital Heidelberg, 69118 Heidelberg, Germany; matthias.gerstner@med.uni-heidelberg.de (M.G.); safak.chasan@med.uni-heidelberg.de (S.C.); 2 Department of Developmental Biology, Center for Medical Biotechnology, University of Duisburg-Essen, 45141 Essen, Germany; ann-christine.severmann@uni-due.de (A.-C.S.); andrea.vortkamp@uni-due.de (A.V.)

**Keywords:** heparan sulfate, proteoglycan, mechanical loading, BMP, chondrocytes, cartilage, tissue engineering, agarose, Bnip3

## Abstract

Osteoarthritis (OA) represents one major cause of disability worldwide still evading efficient pharmacological or cellular therapies. Severe degeneration of extracellular cartilage matrix precedes the loss of mobility and disabling pain perception in affected joints. Recent studies showed that a reduced heparan sulfate (HS) content protects cartilage from degradation in OA-animal models of joint destabilization but the underlying mechanisms remained unclear. We aimed to clarify whether low HS-content alters the mechano-response of chondrocytes and to uncover pathways relevant for HS-related chondro-protection in response to loading. Tissue-engineered cartilage with HS-deficiency was generated from rib chondrocytes of mice carrying a hypomorphic allele of *Exostosin 1* (*Ext1*), one of the main HS-synthesizing enzymes, and wildtype (WT) littermate controls. Engineered cartilage matured for 2 weeks was exposed to cyclic unconfined compression in a bioreactor. The molecular loading response was determined by transcriptome profiling, bioinformatic data processing, and qPCR. HS-deficient chondrocytes expressed 3–6% of WT *Ext1*-mRNA levels. Both groups similarly raised *Sox9*, *Col2a1* and *Acan* levels during maturation. However, HS-deficient chondrocytes synthesized and deposited 50% more GAG/DNA. TGFβ and FGF2-sensitivity of *Ext1^gt/gt^* chondrocytes was similar to WT cells but their response to BMP-stimulation was enhanced. Loading induced similar activation of mechano-sensitive ERK and P38-signaling in WT and HS-reduced chondrocytes. Transcriptome analysis reflected regulation of cell migration as major load-induced biological process with similar stimulation of common (*Fosl1*, *Itgα5*, *Timp1*, and *Ngf*) as well as novel mechano-regulated genes (*Inhba* and *Dhrs9*). Remarkably, only *Ext1*-hypomorphic cartilage responded to loading by an expression signature of negative regulation of apoptosis with pro-apoptotic *Bnip3* being selectively down-regulated. HS-deficiency enhanced BMP-sensitivity, GAG-production and fostered an anti-apoptotic expression signature after loading, all of which may protect cartilage from load-induced erosion.

## 1. Introduction

Articular cartilage covers the bone surfaces of diarthrodial joints and allows for painless articulation at low friction. Its composition of collagen fibers interspersed with negatively charged proteoglycans causes immense swelling pressure making it a highly specialized tissue well adapted to shear forces and load bearing [1]. Compared with other connective tissues, articular cartilage has the lowest extracellular-matrix (ECM) turnover and a very limited intrinsic capacity for healing after injury. Therefore, mechanical injuries and overload are major risk factors for the development of osteoarthritis (OA), a syndrome leading to cartilage degradation and dysfunction of the whole joint. OA can, however, in part be prevented by mechanical stimulation at physiological levels, which is beneficial for the homeostasis of articular cartilage [2,3,4].

The high swelling pressure of cartilage is generated by its high density of negatively charged glycosaminoglycans (GAGs). Main GAGs of articular cartilage are chondroitin sulfate (CS), keratan sulfate (KS), dermatan sulfate (DS), and heparan sulfate (HS), with CS being the most abundant, accounting for 10–35% of cartilage dry weight [5,6]. Remarkably, already during early OA, chondrocytes fail to compensate for the loss of GAGs by enhanced synthesis [7,8,9]. Although the HS-content is 100 times lower than the CS-content in cartilage of porcine articular cartilage, an increasing number of studies suggest a role for HS in OA-development [10,11,12,13,14]. Reduction in HS-synthesizing (*EXT1*) or modifying enzymes (NDST1) [12], deletion of the major HS-bearing proteoglycan Syndecan-4 (SDC4) [13], or ablation of the HS-modification of Perlecan (HSPG2) [14] resulted in chondro-protective effects in mouse models after surgical induction of OA. Recently, enhanced expression of several HS-proteoglycans (HSPG) and HS-modifying enzymes was described in human cartilage, further suggesting a role for HS in osteoarthritis [15]. The mechanisms underlying chondro-protection in HS-reduced cartilage are, however, so far not understood.

Surgical OA-models are based on joint-destabilization leading to mechanical overload of articular cartilage; hence, potential mechanisms protecting HS-reduced cartilage from OA-development may center around the mechano-response of chondrocytes. To understand how the HS-proteoglycan content of cartilage may modulate the mechano-response, one has to consider the mechanisms transducing mechanical forces into the biological loading response. In general, three major mechano-transduction paths have been put forward. First, mechanical forces are directly transduced via force-receptors like integrins and cadherins, which are known to be influenced by HS-bearing SDCs, such as SDC1 and SDC4 [16,17,18,19,20]. Second, cell deformation can activate stretch-induced ion channels like TRPC4, TRPC6, and TRPC7, which can interact with HS-bearing SDCs at the cell surface [21,22,23,24]. Third, deformation of the ECM causes the release of HS-stored signaling factors, changing their bioavailability and receptor interaction as it was demonstrated for FGF2 and TGFβ1 [25,26,27]. Upon loading, HS-stored FGF2 is released from the extracellular matrix, inducing FGFR-signaling and phosphorylation of ERK, a major mechano-response pathway [25,26]. HS-interacting TGFβ1 is stored as latent complex with its pro-domain in the extracellular matrix and can be activated by the release of the inhibiting pro-domain upon force-exertion [27]. In general, effects of HS on sequestration and activation of FGF2, TGFβ, and BMP-signaling are well described [28,29,30]. Overall, HS and HSPGs may influence all three main mechano-transduction paths underlying the mechano-response of chondrocytes, suggesting that a global approach for molecular characterization of the loading response is important.

Mechanical loading studies in vivo are prone to variances in mechanical loading and artifacts due to the animal or tissue harvest after loading. In contrast, in vitro systems can apply force at defined parameters to homogeneous cartilage specimens to obtain consistent and reliable responses to mechanical loading. Bioreactor systems can imitate physiological conditions, allow for setting of defined loading-parameters and for simultaneous application of force to several tested samples at similar amplitude, frequency, and duration [31,32]. Thereby, mechanical variances, as occurring in vivo, can be prevented.

The access to healthy native cartilage tissue is strongly limited and explants show inherent variabilities, like size, shape, cell number, or matrix content. Tissue engineering (TE) of cartilage was constantly improved over decades as a model system to investigate chondrocyte signaling in vitro [33]. Engineered cartilage is available in higher amounts than native tissue and, due to the in vitro generation, more homogeneous between samples compared to cartilage explants. Maturation of TE-constructs is required to obtain engineered cartilage at similar GAG and collagen content compared to native tissue [31]. Different scaffolds emerged for chondrocyte 3D-culture over the years, which provide different advantages in mimicking human cartilage. While collagen sponges offer strong interaction sites leading to pronounced mechano-responses [31,32], hydrogels are often used to induce the spheroidal morphology of chondrocytes and to stabilize their phenotype [34]. Agarose hydrogel has the advantages to provide a defined stiffness and load-transmission and to offer no contact-sites for cells, so the collagens and GAGs involved in cell interaction during loading all derive from the maturing cells themselves. Thus, all interacting proteins and factors in agarose constructs are specific for the cultured cells and offer unique cell type-specific potential for mechano-transduction.

The culture of genetically modified primary chondrocytes from mice enables the generation of mutant 3D-engineered cartilage that can be mechanically challenged. Chondrocytes carrying inactive alleles of specific factors can be cultured and the impact of the deprivation on the mechano-response can be investigated. Chondrocytes with disrupted HS-production are attractive to study the role of the HS-content of cartilage in mechanical loading responses.

Biosynthesis of HS is a multi-step process initiated by the attachment of a linker region to HS-carrying core proteins followed by polymer elongation and the subsequent modification by deacetylation, epimerization, and sulfation [35]. Polymer elongation is catalyzed by the HS-specific glycosyltransferase *EXT1*, which, in complex with *EXT2*, transfers alternating glucuronic acid (GlcA) and N-acetylglucosamine (GlcNAc) monomers to the growing polymer chain. Deletion of *Ext1* in mice leads to full HS-deficiency and is lethal at E8.5 of embryogenesis due to impaired mesoderm formation [36]. In contrast, *Ext1^gt/gt^* (*ext1^Gt(pGT2TMpfs)064Wcs^*) mice [37,38], which carry a hypomorphic allele of *Ext1*, survive until E16.5 when cartilage of the ribs is already well developed. Rib chondrocytes isolated from *Ext1^gt/gt^* mice represent an ideal cell type for investigating the role of HS for chondrocyte signaling in response to loading.

The aim of this study was to clarify whether low HS-content alters the mechano-response of chondrocytes and to uncover HS-related mechanisms relevant for chondro-protection in the context of loading. We hypothesized that low HS-levels will alter the mechano-response of chondrocytes and tested this with our established custom-designed in vitro loading system [31,32] on HS-deficient engineered cartilage generated from rib-cage chondrocytes of *Ext1^gt/gt^* mice [37]. To our knowledge, this is the first study addressing whether HS-deficiency impacts the mechano-response of chondrocytes. A better understanding of the chondro-protective mechanisms in HS-deficient cartilage could provide novel therapy options targeting specific molecular pathways to attenuate OA-development and progression.

## 2. Results

### 2.1. Elevated Glycosaminoglycan Production in HS-Deficient Engineered Cartilage

**Figure 1:**
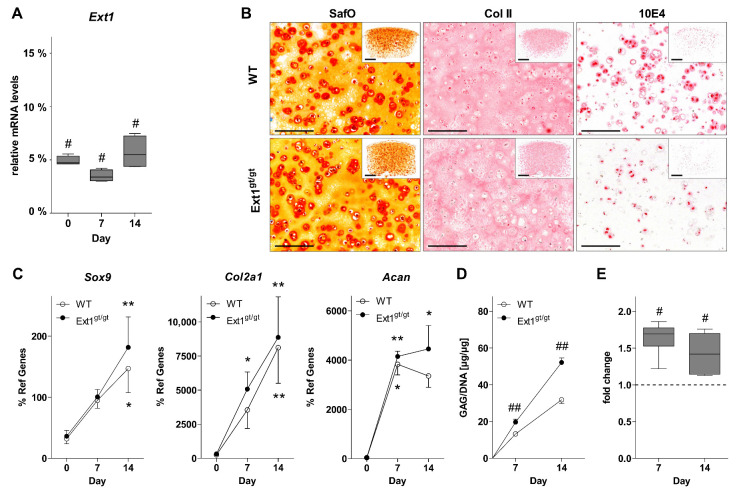
Characterization of *Ext1*-hypomorphic engineered cartilage. Murine chondrocytes from E15.5 *Ext1^gt/gt^* and wildtype (mice were differentiated at 5 × 10^5^ cells per 25 µL agarose construct for indicated time-periods under chondrogenic conditions. (**A**) *Ext1* mRNA levels were determined by RT-qPCR and normalized to reference genes *Rpl19* and *Hprt* with WT samples at the indicated timepoints set to 100%. Data are shown as box plots with each box representing the interquartile range and lines inside the boxes representing the median. Whiskers range from minimum to maximum values (*n* = 4, 4 donors). (**B**) Paraffin sections of engineered cartilage on day 14 were stained with Safranin Orange/Fast Green to visualize glycosaminoglycan (GAG) deposition (SafO, *n* = 22–26, 12 donors) or by immunohistochemistry against Collagen type II (Col II, *n* = 13–16, 6 donors) and the heparan sulfate (HS)-specific epitope 10E4 (10E4, *n* = 9–10, 5 donors). Representative images are shown. Scale bar 200 µm (Inlays 500 µm). (**C**) Expression of differentiation markers during cartilage maturation was determined by RT-qPCR and normalized to reference genes Rpl19 and Hprt (*n* = 5–6, 5–6 donors). (**D**) GAG-content of cartilage tissue determined by 1,9-dimethylmethylene blue (DMMB) assay was normalized to DNA-content (*n* = 6, 3 donors). (**E**) GAG de-novo synthesis was determined by ^35^S-sulfate incorporation over the last 24 h of differentiation culture. Values were normalized to DNA-content and values of WT cartilage were set to 1 (*n* = 6, 3 donors). vs. d0: Kruskal–Wallis * *p* < 0.05, ** *p* < 0.01; *Ext1^gt/gt^* vs. WT: Mann–Whitney U test # *p* < 0.05, ## *p* < 0.01.

For verification of the *Ext1*-hypomorphism of native chondrocytes, *Ext1* mRNA expression was determined in cultured murine rib-cage chondrocytes (mRCs) from *Ext1^gt/gt^* embryos and WT littermates prior to (d0) and on day 7 and 14 of differentiation culture. *Ext1* was expressed at 3–6% of WT levels in *Ext1^gt/gt^* chondrocytes at all tested time points confirming the severely reduced expression of *Ext1* (Figure 1A). After 2 weeks of maturation culture, GAGs and Collagen type II had accumulated in cartilage ECM of both groups according to histology (Figure 1B). Staining intensity of the HS-specific epitope 10E4 was low in *Ext1*-hypomorphic cartilage confirming a strong decrease in HS-levels. Expression of the chondrogenic marker genes *Sox9*, *Col2a1,* and *Acan* rose significantly over time in both groups in a similar manner (Figure 1C). Quantification of proteoglycan deposition revealed a significantly increased GAG/DNA-content in *Ext1^gt/gt^* cartilage to about 150% compared to WT tissue (Figure 1D). Radioactive labeling with ^35^S-sulfate for 24 h before culture termination demonstrated significantly enhanced GAG-synthesis in *Ext1^gt/gt^* chondrocytes on day 7 and 14 compared to WT cells (Figure 1E). Conclusively, HS-deficient *Ext1^gt/gt^* chondrocytes produced significantly more sulfated GAGs and accumulated significantly more proteoglycans in engineered cartilage than WT cells.

### 2.2. Higher BMP-Sensitivity of HS-Reduced Chondrocytes

**Figure 2:**
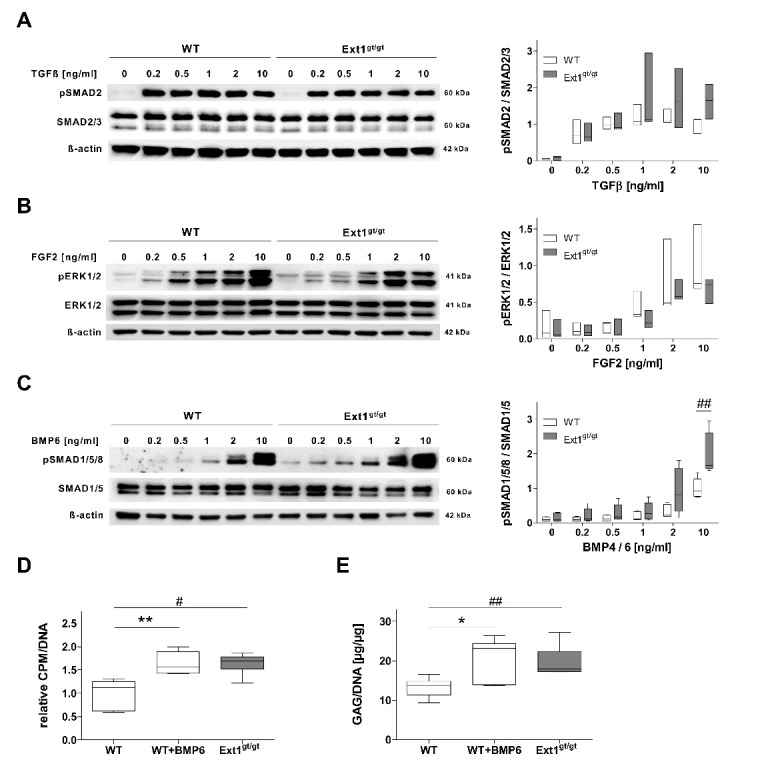
Influence of *Ext1*-hypomorphism on growth factor signaling and GAG-production of engineered cartilage. (**A**–**C**) *Ext1^gt/gt^* and WT chondrocytes were treated in fetal calf serum (FCS)-free monolayer culture with increasing concentrations of indicated growth factors for 3 h. Receptor activation was investigated by Western blot analysis of pSMAD2 /SMAD2/3 (TGFβ, *n* = 3, 3 donors), pERK1/2 /ERK1/2 (FGF2, *n* = 3, 3 donors), and pSMAD1/5/8 /SMAD1/5 (BMP4/6, *n* = 5, 3 donors) levels with β-actin used as a loading control. Phosphorylated forms are considered biologically active. Representative blots are shown. Independent blots were standardized by setting the densitometric intensity of WT cells treated with 10 ng/mL growth factor to 1. (**D**,**E**) Engineered WT cartilage was cultured for 7 days under chondrogenic conditions with or without supplementation of 10 ng/mL BMP6 during the last 96 h (*n* = 6, 3 donors). (**D**) GAG de-novo synthesis was determined by ^35^S-sulfate incorporation over the last 24 h of differentiation culture. Values were normalized to DNA-content and the values of WT controls were set to 1. (**E**) GAG-content of engineered WT cartilage measured by DMMB assay and normalized to DNA-content. Box plots were generated as described in Figure 1. vs. *Ext1^gt/gt^* (#)/untreated (*): Mann–Whitney U test #/* *p* < 0.05, ##/** *p* < 0.01.

Main effects of HS are exerted on the protein level by affecting growth factor signaling at the cell surface [39,40,41,42]. Therefore, we searched for a potential impact of HS-deficiency on TGFβ, FGF, and BMP-pathway activation, which are relevant for cartilage neogenesis. The sensitivity of WT and *Ext1^gt/gt^* chondrocytes to growth-factor stimulation was assessed in short-term monolayer culture in the absence of fetal calf serum (FCS). Chondrocytes were exposed to increasing concentrations of TGFβ, FGF2, BMP6, or BMP4, and receptor activation was determined by Western blot analysis. While for both genotypes TGFβ-stimulation similarly induced pSMAD2 (Figure 2A) and FGF2 similarly induced pERK1/2 (Figure 2B) over the full concentration range, BMP-pathway activation differed between genotypes. At all tested BMP4/6 concentrations, *Ext1^gt/gt^* chondrocytes showed a trend towards a stronger BMP-response compared to WT cells, which reached significance under stimulation with 10 ng/mL BMP (Figure 2C). No altered endogenous *Bmp*-expression in *Ext1^gt/gt^* cartilage samples was recorded compared to WT (Figure S1A). Thus, lower HS-levels enhanced the sensitivity of chondrocytes to pro-chondrogenic BMPs as one possible reason for the enhanced GAG-synthesis observed in HS-deficient cartilage (Figure 1E). To test whether BMP-stimulation would enhance GAG-production in WT chondrocytes, WT specimens were cultured with 10 ng/mL BMP6 during the last 96 h of a 7-day maturation culture. In fact, according to ^35^S-sulfate incorporation on day 7, GAG-synthesis was significantly enhanced (Figure 2D), and the GAG/DNA-content of samples increased significantly to the values of *Ext1^gt/gt^* cartilage (Figure 2E). In conclusion, as BMP-pathway activity was rate limiting for GAG-synthesis in WT chondrocytes, the enhanced BMP-sensitivity of HS-deficient chondrocytes likely leads to the enhanced GAG-production in *Ext1^gt/gt^* cartilage.

### 2.3. Similar Load-Induced Activation of ERK1/2 and P38 Pathways

**Figure 3:**
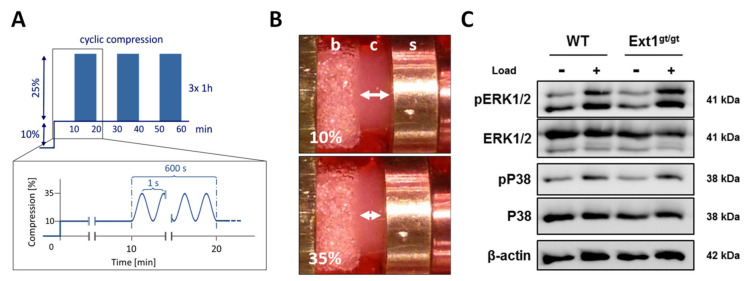
Mechanical loading of engineered cartilage. (**A**) Schematic representation of the dynamic unconfined compression protocol imitating the impact of 3 h of normal walking in 10-min intervals. During a 3-h loading episode, dynamic compression at 1 Hz with an amplitude of 25% was superimposed on 10% static-offset. Nine loading intervals were separated by 10-min breaks. (**B**) Engineered cartilage (“c”) attached to a bone replacement phase (“b”) was fixed in the loading device. A mobile stamp (“s”) applied 10% static compression (upper picture) that was superimposed by 25% dynamic compression (lower picture). (**C**) After 14 days of differentiation culture, *Ext1^gt/gt^* and WT cartilage tissue was exposed to the 3-h loading episode (+) or kept unloaded (-). Specimens were snap frozen immediately at termination of loading. Western blot analysis of active pERK1/2 and total ERK1/2 as well as active pP38 and total P38 levels was performed with β-actin used as a loading control. Representative blots are shown (*n* = 6–9, 5–6 donors).

In order to compare the loading-response of WT and *Ext1*-hypomorphic cartilage, both groups were exposed to a 3-h cyclic unconfined compression episode, which imitates the deformation of human knee-cartilage during 3 h of normal walking in 10-min intervals (Figure 3A,B). Loading of WT and *Ext1*-hypomorphic cartilage pre-cultured for 2 weeks resulted in similar stimulation of the common mechano-response pathways ERK1/2 and P38 according to Western blot analysis (Figure 3C). Other than observed before for human engineered cartilage in a collagen carrier [32], expression of *Bmp2*, *Bmp4,* and *Bmp6* was not altered by loading in both groups (Figure S1B). Furthermore, BMP-signaling was unchanged by loading according to pSMAD1/5/8 levels (data not shown), which is in line with data of a previous study on murine rib chondrocytes in agarose culture [43]. This indicated that lower HS-levels and enhanced BMP-sensitivity had no major impact on the activation of these two common mechano-transduction pathways.

### 2.4. Global Molecular Characterization of the Loading Response

**Figure 4:**
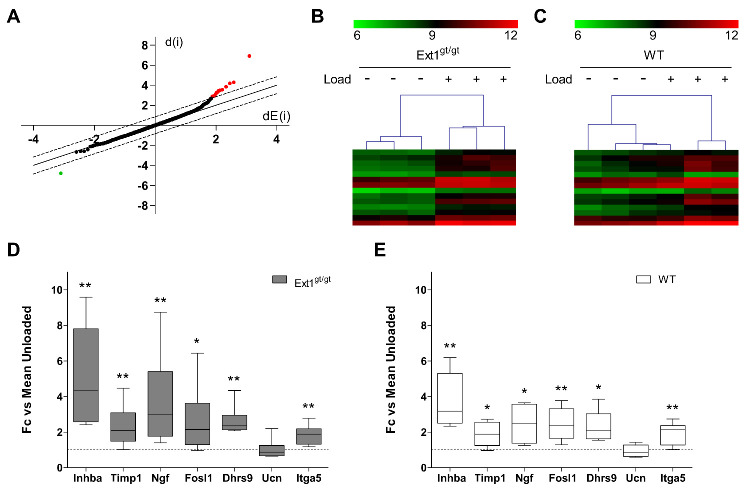
Influence of dynamic compression on global gene expression in HS-deficient and WT cartilage. (**A**–**C**) *Ext1^gt/gt^* and WT cartilage was loaded on day 14 of differentiation culture. Total RNA was isolated from three loaded and non-loaded constructs per group and subjected to whole transcriptome analysis. (**A**) Microarray results depicted as scatter plot following data processing by Significance Analysis of Microarrays (SAM) comparing loaded and non-loaded *Ext1^gt/gt^* engineered cartilage. The observed relative difference d(i) was plotted against the expected relative difference dE(i) for non-loaded and loaded groups. Dashed lines define the difference between d(i) and dE(i) beyond which mRNAs were considered significant at a median false discovery rate (FDR) < 0.05. In red: mRNAs significantly higher for loaded vs. non-loaded group; in green: mRNAs significantly lower in loaded vs. non-loaded group. (**B**) Dendrogram of 14 mRNAs significantly regulated between loaded (+) and non-loaded (-) *Ext1^gt/gt^* samples. (**C**) Dendrogram of WT samples using the 14 hits from the *Ext1^gt/gt^* groups as input. Data are visualized as heatmap of log-2 transformed intensities (*n* = 3, 3 donors). (**D**,**E**) Expression levels of mRNAs (selection from Table 1) in engineered *Ext1^gt/gt^* (**D**) and WT (**E**) cartilage in response to loading. Total RNA was isolated from 6 control and compressed samples per group and subjected to RT-qPCR analysis. Values were normalized to reference genes *Hprt* and *Rpl19* and non-loaded samples were set to 1 (dashed line). Data are shown as box plots as described in Figure 1 (*n* = 6, 5 donors). Loaded vs. non-loaded: Mann–Whitney U test * *p* < 0.05, ** *p* < 0.01.

The overall impact of HS-knockdown on the molecular loading response of engineered cartilage was determined by global transcriptome analysis of mechanically challenged cartilage derived from three independent mRC-pools of *Ext1^gt/gt^* animals and their WT littermates. Comparison of unloaded control samples of WT and HS-deficient tissue confirmed strong knockdown of *Ext1* in *Ext1^gt/gt^* animals and demonstrated that no compensation regarding alteration of gene expression of other HS-synthesis-related genes occurred (Table S2). Bioinformatic processing of data from loaded vs. non-loaded samples by unpaired Significance Analysis of Microarrays (SAM) analysis indicated that only 2 genes were significantly down-regulated by loading in WT cartilage. However, for both, *Exostosin 2* (*Ext2*) and *Smoothened* (*Smo*), this could not be confirmed by RT-qPCR in partially independent samples (data not shown). For the *Ext1^gt/gt^* cartilage group, the same stringent unpaired SAM-analysis delivered 14 genes significantly up-regulated by loading, all of which were induced more than 1.7-fold (Table 1). Only one non-annotated gene was significantly down-regulated (Figure 4A), indicating prevailing cell stimulation. Hierarchical clustering based on the 14 identified hits separated all loaded from non-loaded *Ext1^gt/gt^* samples, underscoring the validity of recognized targets (Figure 4B). Using the same 14 hits for clustering of the WT group showed that one loaded WT sample clustered with the non-loaded specimens, a heterogeneity, which may explain the lack of valid hits by the chosen stringent SAM-analysis in the WT group (Figure 4C).

Next, 7 of the 14 recognized targets were validated by RT-qPCR in partially independent *Ext1*-hypomorphic samples (bold in Table 1). Mechano-induction of 6/7 genes was confirmed (Figure 4D). Among them were common mechano-sensitive genes like *Fosl1*, a member of the Fos family of transcription factors known to shape the early loading response [32,43]; *Itga5*, the main integrin receptor responsible for load transduction in chondrocytes [44,45]; and *Ngf*, (nerve growth factor) and *Timp1,* which are known mechano-responders in chondrocytes [25,46]. Beyond, novel mechano-sensitive genes like *Inhba* and *Dhrs9* were discovered. When mechano-regulation of these genes was next tested by RT-qPCR in the WT group in partially independent sample pairs, a significant induction was found like in HS-deficient cartilage (Figure 4E). This suggested that less stringent bioinformatic analysis of global transcriptome data should be performed as a next step.

### 2.5. Induction of an Anti-Apoptotic Expression Signature in HS-Deficient Cartilage by Loading

**Figure 5:**
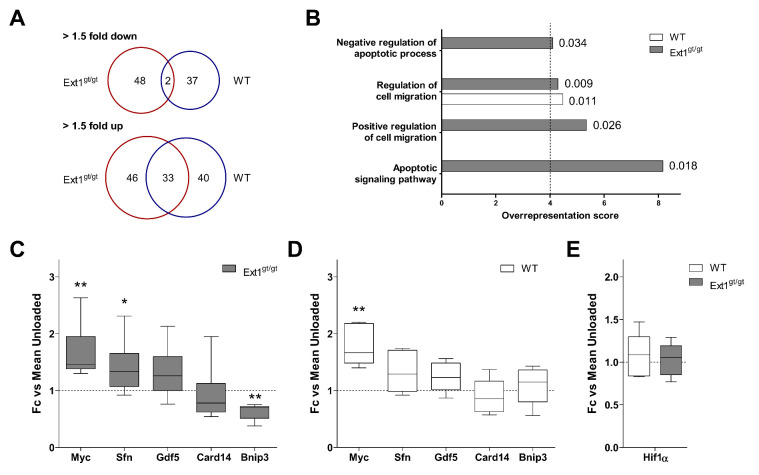
Regulation of mRNA expression (>1.5-fold) in response to loading in HS-deficient and WT cartilage. Microarray data were filtered for genes with a more than 1.5-fold differential expression between loaded and non-loaded samples. (**A**) Venn diagrams are shown, illustrating the differential and overlapping regulation of mRNAs (>1.5-fold) in *Ext1^gt/gt^* vs. WT samples by loading. Numbers in Venn diagrams designate the number of regulated genes per group. (**B**) PANTHER statistical overrepresentation analysis for *Ext1^gt/gt^* and WT cartilage of all genes with a more than 1.5-fold difference in mean expression levels between loaded and non-loaded specimens according to microarray data. Depicted are all Gene Ontology (GO) Biological Process categories with at least 4-fold significant overrepresentation (dashed line) in either genotype according to their overrepresentation score. Numbers behind bars indicate the respective *p*-values. (**C**,**D**) Expression levels of mRNAs (selection from Table 2) in engineered *Ext1^gt/gt^* (**C**) and WT (**D**) cartilage in response to loading. (**E**) Expression levels of *Hif1α* mRNA in *Ext1^gt/gt^* and WT cartilage in response to loading. RT-qPCR analysis was performed as described in Figure 3. Data are shown as box plots as described in Figure 1 (*n* = 6, 5 donors). Loaded vs. non-loaded: Mann–Whitney U test * *p* < 0.05, ** *p* < 0.01.

According to global microarray data, 112 genes were more than 1.5-fold regulated by loading in WT cartilage (Table S3), while 129 responded more than 1.5-fold in HS-deficient cartilage (Table S4). This indicated a slightly higher sensitivity of HS-reduced cartilage to mechanical loading, which may relate to its higher GAG/DNA-content. As illustrated by Venn diagrams, down-regulated genes hardly overlapped between the two groups, while more than 40% of the up-regulated genes were common between WT and HS-deficient cartilage (Figure 5A). To obtain an overview on major biological processes regulated by loading, all genes regulated >1.5-fold in each genotype were subjected to PANTHER statistical overrepresentation analysis for “Gene Ontology Biological Process Complete.” Using a cutoff at 4-fold overrepresentation (Figure 5B), the category “Regulation of cell migration” was overrepresented in both groups (overrepresentation scores 4.29, *p* = 0.009 (*Ext1^gt/gt^*) and 4.47, *p* = 0.011 (WT)). Remarkably, “negative regulation of apoptotic process” (overrepresentation score 4.1, *p* = 0.034), “positive regulation of cell migration” (overrepresentation score 5.34, *p* = 0.026) and “apoptotic signaling pathway” (overrepresentation score 8.16, *p* = 0.018) were significantly overrepresented only in the *Ext1^gt/gt^* group but not in the WT group (Figure 5B).

The category “negative regulation of apoptotic process” consisted of 15 genes regulated by loading in the *Ext1^gt/gt^* group (Table 2). Induction of *Timp1*, *Ngf*, *Dusp1,* and *Itga5* was already recorded by SAM-analysis (Table 1). Beyond, the anti-apoptotic transcription factor *Myc, Sfn,* and the growth factor *Gdf5* were up-regulated, while *Card14*, a caspase-recruitment domain family member, and *Bnip3*, a stress-induced pro-apoptotic BCL2-interacting molecule, were down-regulated. Using RT-qPCR analysis, the significant regulation by loading of *Myc, Sfn,* and *Bnip3* was confirmed in partially independent sample pairs of *Ext1^gt/gt^* cartilage (Figure 5C). Importantly, neither induction of anti-apoptotic *Sfn* nor suppression of pro-apoptotic *Bnip3* occurred after loading in the WT group (Figure 5D), suggesting that their regulation related to the HS-deficiency of *Ext1^gt/gt^* cartilage.

The pro-death factor BNIP3 is pro-apoptotic in the mitochondrial pathway and its expression is increased by HIF1α, which is induced under hypoxic conditions. Since mechanical overloading was previously shown to induce HIF1α in bovine cartilage explants [47], we tested whether our sub-physiological loading protocol affects *Hif1α* expression levels. Gene expression of *Hif1α* was unaltered by loading in WT and *Ext1^gt/gt^* cartilage (Figure 5E), indicating that *Bnip3* down-regulation in *Ext1^gt/gt^* cartilage occurred independent of *Hif1α*. No evidence for load-induced cell death was obtained by a caspase-3 activity assay (data not shown), and mitochondrial activity, detected by an MTT assay performed at the end of loading of day 14 WT samples, reflected no evidence for metabolic stress in response to loading (data not shown). Thus, in the absence of hypoxic and metabolic cell stress, we propose that *Bnip3* may be differentially regulated between the genotypes due to a load-induced physical cell-stress response.

Overall, the data demonstrated that, other than WT cartilage, HS-deficient cartilage gained an anti-apoptotic expression signature after loading and down-regulated expression of the pro-death factor *Bnip3*, an adjustment that may alter the resistance of cells to stress-induced damage and confer protection against negative effects of loading in cartilage tissue.

## 3. Discussion

Loading is an important regulator of cartilage homeostasis, which can have cartilage maintaining functions in the healthy joint but may also support OA-development under permanent improper overstimulation conditions. Reduced HS-expression in articular cartilage decelerated OA-progression after joint destabilization in animal models; the underlying mechanisms, however, remained so far not well understood [12,13,14]. Based on a close connection of HSPGs with main players involved in mechano-transduction, we here speculated that a low HS-content of cartilage may positively influence the loading response of chondrocytes, which consequently would enhance the resistance of cartilage to load-induced degeneration. Our data indicate two likely mechanisms for a better protection of HS-deficient cartilage from deleterious loading: First, a higher BMP-sensitivity of *Ext1^gt/gt^* chondrocytes at maintained TGFβ and FGF2-responsiveness corresponded to an elevated production of proteoglycans compared to WT cells; Second, loading induced an anti-apoptotic expression signature including up-regulation of anti-apoptotic transcription factors and down-regulation of pro-apoptotic *Bnip3*, which may enhance the resistance of cells to stress-induced damage. Altogether, overcompensation of HS-loss by enhanced GAG-production and a more beneficial reaction to mechano-induced cell stress seem likely mechanisms enhancing chondro-protection under mechanical stress leading to the reduced degeneration in HS-deficient cartilage.

Proteoglycans play a central role in governing the poroelastic mechanics, electrically mediated swelling forces and stiffness of cartilage. Thus, they are of primary importance for the response of chondrocytes to mechanical loads. We recently demonstrated a positive correlation between the GAG-content of engineered cartilage and an anabolic response of human chondrocytes to loading after cyclic compression and recognized enhanced silencing of anti-chondrogenic WNT signaling by proteoglycans as one underlying mechanism [31]. Since proteoglycans are flushed out from cartilage under dynamic loading [32], a higher GAG-production capacity of cells will allow for faster replenishment of proteoglycans after mechanical loading to restore the pro-chondrogenic microenvironment and stress-shielding capacity of the tissue. In the current study, we showed that BMP-pathway activity was rate limiting for GAG-production in engineered cartilage since WT chondrocytes enhanced GAG-synthesis under slight stimulation with BMP6, which is in line with the literature [48]. Altogether, this underlines that the here-described enhanced BMP-sensitivity of *Ext1^gt/gt^* chondrocytes is likely responsible for their enhanced GAG-synthesis and may confer stronger chondro-protection in dynamic loading situations.

A connection of HS-deficiency and BMP-signaling has already been addressed in several studies. Matsumoto et al. described in mice carrying a PRX-Cre-driven loss of function allele of *Ext1* that chondrogenesis was delayed and spatial regulation of cartilage condensation was disrupted at maintained pSMAD1/5/8 staining [49]. On the opposite, Huegel et al. reported enhanced BMP-activity in a BMP-reporter assay after treatment of the mesenchymal mouse cell line C3H10T1/2 with the HS-inhibitor Surfen. Furthermore, they observed enhanced chondrogenesis of mouse limb bud cells after Surfen treatment, which was blocked by the BMP-antagonist Noggin [50]. Our study now shows for the first time that mature chondrocytes from rib cages of *Ext1^gt/gt^* animals display an enhanced sensitivity to BMP4- and BMP6-stimulation in culture compared to WT cells. This is associated with and may likely cause elevated GAG-synthesis and GAG-deposition in HS-deficient cartilage. In line, data from Bachvarova et al. indicate that *Ext1^gt/gt^* chondrocytes compensate HS-deficiency by overproduction of chondroitin sulfate, likely attached to aggrecan, a major component of the articular cartilage [10]. Increased cartilage matrix formation also occurs in affected tissue of mouse mutants mimicking Hereditary Multiple Exostoses (HME), a disease linked to aberrant *Ext1*-expression [51,52,53]. Interestingly, in HME, local HS-deficiency co-localizes with ectopic BMP-signaling and excessive cartilage growth in developing long bones to so-called osteochondromas [49,50]. Thus, the enhanced BMP4/6-sensitivity and GAG-production discovered here for *Ext1^gt/gt^* rib chondrocytes is in line with findings on pharmacological or structural HS-depletion in mesenchymal progenitors and on HS-deficient osteochondroma cells in animal models of HME. Mundy et al. described restraining effects of HS on BMP-ligand availability and BMPR dynamics in Ad-293 cells [40]. Since BMP-expression was similar between WT and *Ext1^gt/gt^* cartilage and there was no exogenous BMP in our serum-free differentiation medium, we propose that altered availability of cell-produced BMPs and alterations in ligand-receptor dynamics are likely mechanisms explaining enhanced GAG-production in HS-deficient chondrocytes.

Integrins are main players in sensing of mechanical signals, in regulating cell–matrix interaction and in transducing the signals along the MAPK pathway resulting in ERK-activation [54]. Fibronectin-binding ITGA5 is considered the main integrin mechano-receptor of chondrocytes [44,45] and its expression is known to be up-regulated in response to loading [55]. In line with its basic role in mechano-transduction and rapid adaptation to mechanical challenge, we observed a similar induction of *Itga5* expression in WT and mutant cartilage. Furthermore, induction of the same immediate early response genes like *Fosl1* and *Ngf* and a comparable activation of the mechano-sensitive pathways ERK1/2 and P38 in both groups was fostered in response to loading. This suggests that basic mechanisms of force perception via integrin receptors and translation of physical forces into common mechano-transduction pathways are little affected by reduced HS-levels.

The strong mechano-induction of *Inhba* (>4-fold), a subunit of activin/inhibin complexes belonging to the TGFβ-superfamily, is a novel finding of our study. Two subunits of *Inhba* form the homodimer Activin A, which signals through canonical ALK4-ACVR2 receptor complexes activating the transcription factors SMAD2 and SMAD3. Activin A has a strong affinity to type 2 receptors, similar to certain BMPs. One important way, how activin A regulates cell behavior, is by antagonizing BMP-ACVR2A/ACVR2B/ALK2 signaling [56]. In WT chondrocytes, BMP-sensitivity, and proteoglycan synthesis were lower than in *Ext1^gt/gt^* chondrocytes. Therefore, it is tempting to speculate that a potential further reduction in BMP-activity due to load-induced *Inhba* could be more critical regarding compensation of proteoglycan loss in WT cells than in HS-deficient chondrocytes. Similar up-regulation of *Inhba* by loading could therefore impact *Ext1^gt/gt^* chondrocytes differentially than WT cells, a possibility that should further be investigated in follow-up studies.

One important novelty of our study is that mechanical loading promoted an overall anti-apoptotic expression signature only in HS-deficient cartilage. Anti-apoptotic genes like the transcription factors *Jun* and *Myc* as well as *Sfn, Ngf*, and *Gdf5* were up-regulated in response to loading, while pro-apoptotic Bcl-2 interacting protein 3 (*Bnip3*) was suppressed. Vernon et al. described anti-apoptotic effects for sub-physiological loading of native cartilage plugs after compaction injury and suggested that therapeutic exercises could be designed to deliver sub-physiological loading to the cartilage, thereby minimizing injury [57]. Lee et al. demonstrated that shear stress-induced nitric oxide (NO) is associated with changes in apoptotic regulatory factors that alter chondrocyte metabolism and may contribute to joint degeneration [58]. NO is an important signaling molecule known to be produced in response to mechanical loading [58,59]. Remarkably, in hepatocytes, NO-production suppressed the expression of BNIP3 as part of an anti-apoptotic cell response [60]. Thus, it is tempting to speculate that differential NO-production or NO-downstream signaling between WT and HS-deficient cartilage in response to loading may explain the discrepant *Bnip3* regulation discovered in this study, an important question that should be answered in future studies.

## 4. Materials and Methods

### 4.1. Transgenic Mice

Mice were kept and bred according to the institutional guidelines of the University of Duisburg-Essen and the University Hospital Essen, specifically approved by the animal welfare officer of the University of Duisburg-Essen. Animal care was approved by the city of Essen (Az: 32–2-11–80-71/ 348) in accordance with § 11 (1) 1a of the “Tierschutzgesetz”. Work with transgenic animals was approved by the “Bezirksregierung Duesseldorf” (Az: 53.02.01-D-1.55/12, Anlagen-Nr. 1464) in accordance with § 8 Abs. 4 Satz 2 GenTG of the “Gentechnikgesetz”.

Noon of the day, when a vaginal plug was detected, was defined as embryonic day 0.5 (E0.5) of timed pregnancies. *Ext1^gt/gt^* mice were bred by mating heterozygous *Ext1^gt^* (*ext1^Gt(pGT2TMpfs)064Wcs^*) mice that were maintained on a C57Bl/6 J background [37]. Genotyping was performed by genomic PCR of tail biopsies as described elsewhere [10].

### 4.2. Cell Isolation and Culture

Primary murine rib-cage chondrocytes (mRCs) were isolated from E15.5 embryonic mice as described elsewhere with adaptations [61]. In short, single ribs were dissected from rib cages in PBS, cleaned manually from perichondrium tissue under the microscope and digested in 2 mg/mL collagenase-B (Worthington, Lakewood, NJ, USA) in expansion medium (DMEM, 1 g/L glucose, 10% FCS, 100 U/mL penicillin, and 100 µg/mL streptomycin) at 37 °C for 3 h with vigorous resuspension every 30 min. mRCs were washed in PBS and plated at 5000 cells/cm^2^ in culture flasks and expanded for 6–7 days in expansion medium at 37 °C, 5% CO_2_.

For investigating growth factor signaling, 80,000 cells/well were plated in 12-well plates (21,000 cells/cm^2^) and cultured for 2 days in expansion medium. Cells were rinsed in PBS and stimulated with growth factors for 3 h in DMEM without FCS. After rinsing in PBS, cells were lysed in 150 µL ice-cold RIPA buffer (50 mM Tris-HCl, 1% Triton X-100, 0.1% SDS, 12 mM sodium deoxycholate, 150 mM NaCl, 1 mM EDTA, and 2 mM β-glycerophosphate) with PefaBloc (Merck, Darmstadt, Germany). Cell debris was removed by centrifugation at 13,000 rcf for 10 min before Western blot analysis as described below.

### 4.3. Generation and Culture of Engineered Cartilage

At the end of expansion, mRCs were trypsinized and resuspended in chondrogenic medium (DMEM, 4.5 g/L glucose, 0.1 mM dexamethasone, 0.17 mM ascorbic acid-2 phosphate, 1 mM sodium pyruvate, 0.35 mM proline, 100 U/mL penicillin, and 100 µg/mL streptomycin) containing 1% ITS+ Premix (Corning, NY, USA) and 10 ng/mL TGFβ to obtain a concentration of 6 × 10^7^ cells/mL. Two volumes of melted 3% low-melt agarose (peqGOLD, 35–2020, VWR Peqlab, Darmstadt, Germany) at 39 °C were added to the pre-warmed cell suspension to obtain a final concentration of 2 × 10^7^ cells/mL in 2% agarose. Briefly, 25 µL suspension containing 5 × 10^5^ cells were casted into custom-made silicone molds (4 mm in diameter, 2 mm in height). After solidification at room temperature for 5 min, agarose disks were cultured in 1.5 mL chondrogenic medium for 2 days and then attached with fibrin gel to custom-made porous glass blocks (ROBU, Hattert, Germany) having approximately 36% pore volume and 40–100 µm pore size, which measured 4 mm × 4 mm × 6 mm. Glass carriers served as a bone-replacement phase to allow medium exchange during culture and loading. Biphasic constructs were cultured for indicated time-periods with medium change every 2–3 days. Where indicated, chondrogenic medium was supplemented with growth factors at the given concentrations.

### 4.4. RNA Isolation and RT-qPCR

Cartilage constructs were snap frozen at harvest and halved before RNA isolation. Total RNA was isolated using the QIAquick Gel Extraction Kit (QIAGEN, Hilden, Germany) with an adapted protocol. In short, half a construct supplemented with 500 µL GQ Buffer was disintegrated with a Polytron homogenizer and incubated at 50 °C for 25 min while shaking to dissolve the agarose carrier and lyse cellular membranes. Briefly, 166 µL 2-propanol was added and RNA was purified by silica-membrane columns. cDNA was transcribed using Omniscript RT Kit (Qiagen) with oligo(dT) primers. Quantitative PCR (qPCR) was performed with SYBR green (Thermo Fisher, Waltham, MA, USA) using primers listed in Table S1. Genes were considered expressed only if agarose gel electrophoresis of PCR reactions revealed distinct bands at the correct running height and only single melting peaks at the correct temperature appeared during qPCR. Expression levels were calculated using the ΔCt method vs. the arithmetic mean of two reference genes, *Hprt* and *Rpl19*. Depicted % reference genes (% Ref Genes) was calculated as 1.8^(−ΔCt) × 100.

### 4.5. Histology

Cartilage constructs were fixed in 4% formaldehyde and dehydrated in increasing concentrations of 2-propanol and acetone before paraffin-embedding. For histology, 5 µm sections were cut, de-paraffinized, and rehydrated. Sulfated glycosaminoglycans (GAGs) were stained with 0.2% (m/V) Safranin O (Fluka, Sigma Aldrich, St. Louis, MO, USA) in 1% acetic acid and 0.04% (m/V) Certistain Fast Green (Merck) in 0.2% acetic acid as counter-staining. For immunohistochemistry (IHC) against Collagen type II and heparan sulfate (10E4-epitope), rehydrated sections were subsequently digested with 4 mg/mL hyaluronidase in PBS, pH 5.5, at 37 °C for 15 min for antigen retrieval. Collagen type II-stained sections were additionally digested with 1 mg/mL pronase in PBS, pH 7.4, at 37 °C for 30 min. Blocking in 5% BSA (Sigma Aldrich) was followed by incubation with primary antibody against human Collagen type II (1:1000, ICN Biomedicals, Eschwege, Germany, clone II-4C11) or an HS-specific epitope (1:200, AMS Biotechnology, Abingdon, UK, clone F58-10E4). The BrightVision Poly-AP-Anti Ms/Rb IgG, one component kit (ImmunoLogik, Duiven, The Netherlands) and ImmPACT Vector Red (Vector, Peterborough, UK) as alkaline phosphatase substrate were used for detection and Collagen type II-stained sections were counterstained with Mayer’s Hematoxylin. Slides were permanently mounted with Neo-Mount (SafO, Merck) or Aquatex (IHC, Merck) before light microscopy.

### 4.6. GAG and DNA-Quantification

Constructs were digested with 0.5 mg/mL Proteinase K (Thermo Fisher) in digestion buffer (50 mM Tris-HCl, 1 mM CaCl_2_, pH 8) at 65 °C while shaking overnight. Concentrations of GAGs within the digests were quantified by the 1,9-dimethylmethylene blue (DMMB) assay using chondroitin-sulfate A (Sigma Aldrich) as a standard [62]. DNA was quantified with Quant-iT™-PicoGreen^®^ (Life Technologies, Thermo Fisher). The GAG-content was normalized to the DNA content.

### 4.7. GAG-Synthesis

De-novo synthesis of highly sulfated GAGs was determined by ^35^S-sulfate incorporation. Tissue-engineered cartilage was posed on a nylon mesh in a 48-well plate to allow full contact to 500 µL chondrogenic medium supplemented with 4 μCi ^35^SO_4_ (Hartmann Analytic, Braunschweig, Germany, ARS0105). Label incorporation was performed in the incubator at 37 °C, 5% CO_2_ for 24 h. After 5 washing steps in 500 μL 1 mM Na_2_SO_4_ in PBS for 20 min while shaking, samples were digested with Proteinase K as described for GAG and DNA-quantification. Incorporated label was quantified by β-scintillation counting using the program Winspectral. Radioactivity was normalized to DNA-content determined as described above.

### 4.8. Mechanical Loading

The last change of chondrogenic medium was performed 24 h prior to mechanical stimulation. Tissue-engineered cartilage together with the medium was transferred into our custom-built bioreactor system 2 h before loading [32]. Cartilage was pre-compressed at 10% of its thickness (static-offset) to maintain contact to the piston throughout compression. For 3 h, 10-min intervals of static offset were intermitted by superimposition of 25% dynamic compression at 1 Hz for 10 min. Free-swelling controls were kept in the same device without loading. Samples were snap frozen directly after the end of the last dynamic loading interval and stored at −80 °C until processed.

### 4.9. Protein Lysates and Western Blot

Half a construct was disintegrated in 75 µL PhosphoSafe Extraction Reagent (Merck Millipore) supplemented with 1 mM Pefabloc SC (Merck) using a mixer mill (Retsch, Haan, Germany) at 30 Hz for 2 × 2 min and cell debris was removed by centrifugation at 13,000 rcf for 20 min. Proteins were separated by denaturing SDS-gel electrophoresis in 10% poly-acrylamide gels before blotting on a nitrocellulose membrane (GE Healthcare, Berlin, Germany). To stain proteins of different heights in parallel, the membrane was cut at 50 kDa. Blocking in 5% milk powder in 0.05% TBST for 1 h was followed by incubation with primary antibody against *p*-ERK1/2 (1:200, Santa Cruz, Dallas, TX, US, sc-7383, clone E-4), ERK1/2 (1:1000, Cell Signaling Technology, Danvers, MA, US, 9102), pP38 (1:1000, Cell Signaling Technology, 4511, clone D3F9), P38 (1:1000, Cell Signaling Technology, 9212), pSMAD2 (1:250, Cell Signaling Technology, 3108, clone 138D4), SMAD2/3 (1:1000, Cell Signaling Technology, 8685, clone D7G7), pSMAD1/5/8 (1:250, Cell Signaling Technology, 13820S), SMAD1 (1:500, Abcam, Cambridge, UK, Ab33902, clone EP565Y), SMAD5 (1:1000, Abcam, Ab40771, clone EP619Y), or β-actin (1:10,000, GeneTex, Irvine, CA, US, GTX26276, clone AC-15) in 5% milk overnight at 4 °C. Membranes were washed 3 × 5 min and incubated with secondary antibodies in 5% milk that were peroxidase-conjugated goat anti-mouse (1:5000, Jackson ImmunoResearch, Cambridge, UK, 111-035-046) or goat anti-rabbit (1:10,000, Jackson ImmunoResearch, 115-035-071) IgGs for 2 h at room temperature. Membranes were washed 3 × 5 min and the Western Bright Chemiluminescence Substrate kit (Biozym, Hessisch Oldendorf, Germany) was used for detection in the Fusion-SL 3500 WL imaging system (VWR Peqlab).

### 4.10. Caspase 3 Activation and MTT Assay

For the caspase assay, cartilage specimens were incubated with 0.1 µg/mL fluorescein diacetate in PBS for 5 min at 37 °C, snap frozen, and lysed (50 mM HEPES, 1.5% CHAPS, 2 mM DTT). Briefly, 100 µL lysate was incubated with 100 µL 20 µM Ac-DEVD-AMC (Biomol) in assay buffer (20 mM HEPES, 2 mM EDTA, 0.1% CHAPS, and 2 mM DTT). Fluorescence of cleaved substrate was normalized to fluorescein. For the MTT-assay, 0.2 mg/mL MTT (3-(4,5-Dimethylthiazol-2-yl)-2,5-Diphenyltetrazolium Bromide) was supplemented to the medium for 2 h. MTT-formazan was solubilized with 2-propanol and quantified photometrically.

### 4.11. Microarray Analysis

Total RNA was isolated as described above and subjected to mRNA expression analysis using the Clariom^TM^ S Assay, mouse (Affymetrix/Thermo) detecting expression levels of >20,000 mRNAs. RNA quality control, labeling, array hybridization, and microarray scanning were performed at the Genomics and Proteomics Core Facility at the German Cancer Research Center, Heidelberg. Intensity values from cDNA array analysis were quantile-normalized, log2-transformed, and analyzed in MultiExperiment Viewer 4.9.0 (TM4 Microarray-Software-Suite). Venn diagrams were calculated using the Bioinformatics & Evolutionary Genomics website: http://bioinformatics.psb.ugent.be/webtools/Venn/ (accessed on 20 December 2019). PANTHER overrepresentation analysis was performed for the PANTHER Gene Ontology (GO)-Biological Process Complete category, submitting the list of all genes with a more than 1.5-fold differential mean expression between loaded and non-loaded samples to the PANTHER software (Version 15.0, released 2020-02-14; Mi et al. 2019). Overrepresentation was tested by Fisher’s exact test with Bonferroni correction for multiple testing using the *Mus musculus* database (DOI:10.5281/zenodo.4081749 Released 2020-10-09) as reference list. Microarray data were further analyzed by unpaired Significance Analysis of Microarrays (SAM) [63]. For multiple testing in SAM-analysis, correction with the median false discovery rate (FDR) set to <0.05 was applied. Each engineered cartilage construct was considered an independent biological sample.

### 4.12. Statistics

Timelines are presented as means with SEM. For multiple comparisons in time courses, Kruskal–Wallis with Dunn’s multiple comparison test was performed. Group comparisons are depicted as box plots visualizing the interquartile range around the median (line) with whiskers ranging from minimum to maximum values. Statistical significance between groups was tested by non-parametric Mann–Whitney U test with Bonferroni correction in case of multiple comparisons. For all comparisons, the mean of all controls was set to 1. A probability value of *p* < 0.05 was considered statistically significant. Data analysis was performed using GraphPad Prism 9.

## 5. Conclusions

Altogether, our data identified two likely mechanisms for an improved protection of HS-deficient cartilage from load-induced degeneration: superior GAG-production likely due to an enhanced BMP-sensitivity of HS-deficient chondrocytes and an anti-apoptotic expression signature gained by loading, which we consider as a more favorable downstream mechano-response compared to WT cartilage. Beyond the basic value of our data and new ideas about diagnostic and therapeutic targets in HS-relevant pathways, better knowledge of HS-related chondro-protection may allow to design pharmacological treatments to reduce HS-levels and combine them with therapeutic exercises to deliver sub-physiological loading to the cartilage in early OA or after cartilage injury.

## Figures and Tables

**Table 1:** Mean microarray expression levels of mRNAs differentially expressed in loaded and non-loaded *Ext1^gt/gt^* samples according to Significance Analysis of Microarrays.

Gene Symbol	Gene Name	Mean Intensities		
		Ctrl	Load	Fold Change
***Inhba***	Inhibin beta-A	253	1023	4.04
*Gjb4*	Gap junction protein, beta 4	217	763	3.52
*Gprc5a*	G protein-coupled receptor C, 5, a	245	760	3.11
***Timp1***	Tissue inhibitor of MMPs 1	988	2518	2.55
*Cd44*	CD44 antigen	244	590	2.42
***Ngf***	Nerve growth factor	336	811	2.41
*Plaur*	PLG-activator, urokinase receptor	179	409	2.29
*Srxn1*	Sulfiredoxin 1	224	469	2.09
*Dusp1*	Dual specificity phosphatase 1	1720	3590	2.09
***Fosl1***	Fos-like antigen 1	255	528	2.07
***Dhrs9***	Dehydrogenase/reductase 9	101	209	2.06
*Nt5e*	5′-nucleotidase, ecto	593	1214	2.05
***Ucn***	Urocortin	151	283	1.87
***Itga5***	Integrin alpha 5	1404	2494	1.78

bold: genes tested by RT-qPCR.

**Table 2:** Mean microarray expression levels of the genes recorded in the PANTHER “Negative Regulation of Apoptotic Process” category in loaded and non-loaded *Ext1^gt/gt^* samples.

		Mean Intensities		
Gene Symbol	Gene Name	Ctrl	Load	Fold Change
Up-regulated				
Timp1 *	Tissue inhibitor of MMPs 1	988	2518	2.55
Ngf *	Nerve growth factor	336	811	2.41
*Dusp1*	Dual specificity phosphatase 1	1720	3590	2.09
*Ptgs2*	Prostaglandin G/H synthase 2	261	494	1.90
***Ucn***	Urocortin	151	283	1.87
***Smo***	Smoothened, frizzled class receptor	189	336	1.78
Itga5 *	Integrin alpha 5	1404	2494	1.78
***Myc***	Myc proto-oncogene protein	263	452	1.72
*Spry2*	Sprouty homolog 2	132	207	1.56
*Jun*	Jun proto-oncogene	406	625	1.54
***Sfn***	Stratifin, alias 14–3-3 protein sigma	155	237	1.53
***Gdf5***	Growth differentiation factor 5	376	567	1.51
Down-regulated				
***Bnip3***	BCL2 interacting protein 3	1073	663	−1.62
*Irs2*	Insulin receptor substrate 2	321	199	−1.61
***Card14***	Caspase recruitment domain family, member 14	134	88	−1.52

bold: genes tested by RT-qPCR, * confirmed genes from SAM-analysis.

## Data Availability

The cDNA microarray data described in this manuscript can be found on: https://www.ebi.ac.uk/arrayexpress/E-MTAB-10258.

## Supplementary Materials

The following are available online at https://www.mdpi.com/article/10.3390/ijms22073726/s1, Figure S1: Expression levels of selected *Bmps* during cartilage maturation and in response to loading in HS-deficient and WT cartilage, Table S1: qPCR primer list, Table S2: Expression of selected genes related to heparan sulfate synthesis in WT and *Ext1^gt/gt^*-engineered cartilage according to cDNA array analysis, Table S3: Expression of genes regulated >1.5-fold in WT cartilage in response to loading, Table S4: Expression of genes regulated >1.5-fold in *Ext1^gt/gt^* cartilage in response to loading.
