# Supplementary material for: Heparan Sulfate Deficiency in Cartilage: Enhanced BMP-Sensitivity, Proteoglycan Production and an Anti-Apoptotic Expression Signature after Loading
Source: Int J Mol Sci. 2021 Apr 2;22(7):3726. doi: 10.3390/ijms22073726 (PMC8038223; doi:10.3390/ijms22073726)
Supplement: Supplementary file 1 [file ijms-22-03726-s001.pdf]

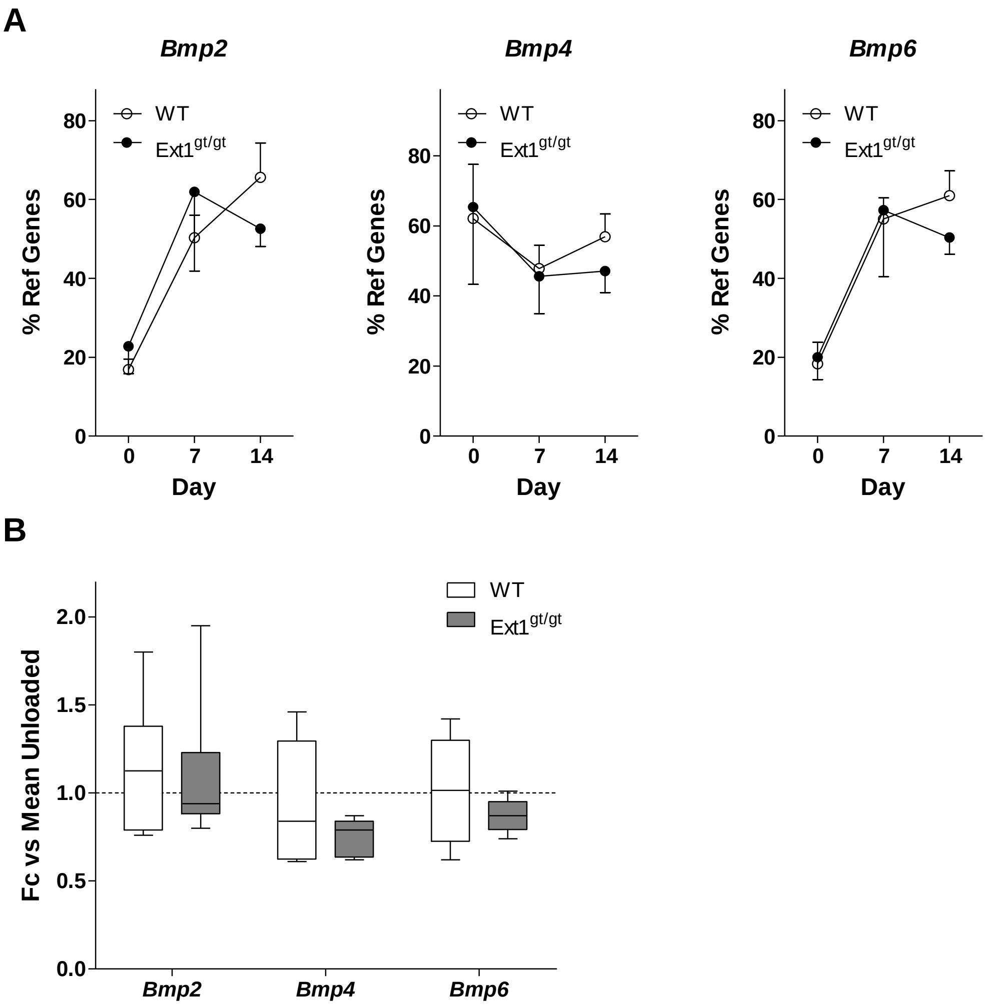

Figure S1 Expression levels of selected *Bmps* during cartilage maturation and in response to loading in HS-deficient and WT cartilage. (A) Expression levels of indicated *Bmps* during cartilage maturation. RT-qPCR analysis was performed as described in Fig 1 (n = 4-7, 4-6 donors). (B) Expression levels of indicated *Bmps* in engineered $Ext1^{gt/gt}$ and WT cartilage in response to loading on day 14 of differentiation culture. RT-qPCR analysis was performed as described in Fig 4. Data are shown as box plots as described in Fig 1 (n = 6, 5 donors). *vs* d0: Kruskal-Wallis (no significance); $Ext1^{gt/gt}$ vs WT: Mann-Whitney U test (no significance).

Table S1 qPCR primer list.

Gene	Primer Forward	Primer Reverse
Acan	5´ - CCAGCCTACACCCCAGTG - 3´	5´ - CAGAGGGGCTTTCTGTGTGT - 3´
Bnip3	5´ - CCTGTCGCAGTTGGGTTC - 3´	5´ - GAAGTGCAGTTCTACCCAGGAG - 3´
Card14	5´ - CCAGGAGAAGGCACAGAAGG - 3´	5´ - TGCAGTGACAGGTTCAGCAT - 3´
Col2a1	5´ - GTCCTTCTGGCCCTAGAGGT - 3´	5´ - TGTTTCTCCTGAGCGTCCA - 3´
Dhrs9	5´ - ATTTGGTGGAGGGGGCTA - 3´	5´ - TGCACACCAAAAGCTTTCAT - 3´
Ext1	5´ - CTGGCCAACTGTGAGGACAT - 3´	5´ - CGGGAAGTCTGTCCCATCAT - 3´
Fosl1	5´ - GAGACGCGAGCGGAACAAG - 3´	5´ - AATTTGTCGGTCTCCGCCTG - 3´
Gdf5	5´ - CAGTCAGTTGTGCGGGAGAA - 3´	5´ - TGAGTGTCTTGAAGTCCGCC - 3´
Hprt	5´ - TGATAGATCCATTCCTATGACTGTAGA - 3´	5´ - AAGACATTCTTTCCAGTTAAAGTTGAG - 3´
Inhba	5´ - GGGAGTGATCCCTGGAAAC - 3´	5´ - CAGCAAATTCTCTTTCGGGTCC - 3´
Itga5	5´ - GCCATTTAGCCTTCAGTGTGA - 3´	5´ - GTAGGGGAGGGAACGTTTGA - 3´
Myc	5´ - AGCGACTCTGAAGAAGAGCAAG - 3´	5´ - AGTTGTGCTGGTGAGTGGAG - 3´
Ngf	5´ - GGGGAGCGCATCGAGTTTT - 3´	5´ - CCTCACTGCGGCCAGTATAG - 3´
Rpl19	5´ - TCATCCAGGTCACCTTCTCA - 3´	5´ - CTCGTTGCCGGAAAAACA - 3´
Sfn	5´ - ACAGGCCGAACGGTATGAAG - 3´	5´ - GTACTCTTTCACCTCGGGGC - 3´
Sox9	5´ - CAGCAAGACTCTGGGCAAG - 3´	5´ - ATCGGGGTGGTCTTCCTTGT - 3´
Timp1	5´ - GCAAAGAGCTTTCTCAAAGACC - 3´	5´ - AGGGATAGATAAACAGGGAAACACT - 3´
Ucn	5´ - ACTGTCCATCGACCTCACCTTCCA - 3´	5´ - ACTGAGACAGCTCCGGTTGTGC - 3´

Table S2 Expression of selected genes related to heparan sulfate synthesis in WT and *Ext1*$^{gt/gt}$ engineered cartilage according to cDNA array analysis.

		Mean Intensities		
**Gene Symbol**	**Gene Name**	**WT**	**Ext1$^{gt/gt}$**	**Fold Change**
Xylt1	xylosyltransferase 1	1761	1845	1.05
Xylt2	xylosyltransferase 2	165	187	1.14
Ext1	**exostoses (multiple) 1**	**887**	**123**	**0.14**
Ext2	exostoses (multiple) 2	520	413	0.79
Ndst1	N-deacetylase/N-sulfotransferase (heparan glucosaminyl) 1	624	514	0.82
Ndst2	N-deacetylase/N-sulfotransferase (heparan glucosaminyl) 2	249	242	0.97
Ndst3	N-deacetylase/N-sulfotransferase (heparan glucosaminyl) 3	61	67	1.10
Ndst4	N-deacetylase/N-sulfotransferase (heparan glucosaminyl) 4	66	70	1.06
Glce	glucuronyl C5-epimerase	442	486	1.10
Hs2st1	heparan sulfate 2-O-sulfotransferase 1	229	242	1.06
Hs6st3	heparan sulfate 6-O-sulfotransferase 3	132	119	0.91
Hs3st6	heparan sulfate (glucosamine) 3-O-sulfotransferase 6	105	87	0.82
Hs6st1	heparan sulfate 6-O-sulfotransferase 1	143	120	0.84
Hs6st2	heparan sulfate 6-O-sulfotransferase 2	94	100	1.06
Hs3st5	heparan sulfate (glucosamine) 3-O-sulfotransferase 5	104	108	1.04
Hs3st3a1	heparan sulfate (glucosamine) 3-O-sulfotransferase 3A1	164	157	0.96
Hs3st3b1	heparan sulfate (glucosamine) 3-O-sulfotransferase 3B1	242	243	1.01
Hs3st1	heparan sulfate (glucosamine) 3-O-sulfotransferase 1	166	186	1.12
Hs3st2	heparan sulfate (glucosamine) 3-O-sulfotransferase 2	243	259	1.07
Hs3st4	heparan sulfate (glucosamine) 3-O-sulfotransferase 4	103	100	0.97
Sulf1	sulfatase 1	350	370	1.06
Hpse	heparanase	76	79	1.04
Hpse2	heparanase 2	81	76	0.94
Papss1	3-phosphoadenosine 5-phosphosulfate synthase 1	485	484	1.00
Papss2	3-phosphoadenosine 5-phosphosulfate synthase 2	5924	5945	1.00
Extl1	exostoses (multiple)-like 1	443	420	0.95
Extl2	exostoses (multiple)-like 2	468	443	0.95
Extl3	exostoses (multiple)-like 3	526	489	0.93

Table S3 Expression of genes regulated >1.5-fold in WT cartilage in response to loading.

Gene Symbol	Gene Name	Mean Intensities Ctrl	Mean Intensities Load	Fold Change
Up-regulated				
Inhba	inhibin beta-A	409	1149	2.81
Gprc5a	G protein-coupled receptor, family C, group 5, member A	361	943	2.61
Gjb4	gap junction protein, beta 4	352	858	2.44
Dusp1	dual specificity phosphatase 1	1562	3709	2.37
Ptgs2	prostaglandin-endoperoxide synthase 2	240	546	2.28
Cd44	CD44 antigen	346	779	2.26
Timp1	tissue inhibitor of metalloproteinase 1	1159	2487	2.15
Svs4	seminal vesicle secretory protein 4	54	113	2.10
Srsf2	serine	305	629	2.06
Plaur	plasminogen activator, urokinase receptor	219	449	2.05
Phlda1	pleckstrin homology-like domain, family A, member 1	351	647	1.84
Hbegf	heparin-binding EGF-like growth factor	415	761	1.83
Jun	jun proto-oncogene	329	602	1.83
Snx16	sorting nexin 16	74	134	1.80
Ngf	nerve growth factor	504	888	1.76
Slc20a1	solute carrier family 20, member 1	588	1031	1.75
Hmga1-rs1	high mobility group AT-hook I, related sequence 1	367	641	1.75
Tnfaip6	tumor necrosis factor alpha induced protein 6	251	431	1.72
Pxylp1	2-phosphoxylose phosphatase 1	170	290	1.71
Fosl1	fos-like antigen 1	272	461	1.70
Prss46	protease, serine 46	195	329	1.69
Defb8	defensin beta 8	133	223	1.67
Ccdc64b	coiled-coil domain containing 64B	80	133	1.66
Hoxb1	homeobox B1	81	134	1.66
Sema7a	semaphorin 7A	159	263	1.66
Dusp8	dual specificity phosphatase 8	276	456	1.65
Bpifc	BPI fold containing family C	124	203	1.64
Hmga1	high mobility group AT-hook 1	299	488	1.63
Emp1	epithelial membrane protein 1	1471	2392	1.63
Itga5	integrin alpha 5 (fibronectin receptor alpha)	1549	2516	1.62
Dusp10	dual specificity phosphatase 10	127	205	1.61
Krt6a	keratin 6A	138	220	1.60
Nt5e	5 nucleotidase, ecto	766	1220	1.59
Gm9869	predicted gene 9869 [Source:MGI Symbol;Acc:MGI:3642719]	76	120	1.59
Pde1b	phosphodiesterase 1B, Ca2+-calmodulin dependent	386	614	1.59
Nr4a3	nuclear receptor subfamily 4, group A, member 3	107	170	1.59
Apoe	apolipoprotein E	368	577	1.57
Flnc	filamin C, gamma	156	244	1.57
Rcan1	regulator of calcineurin 1	194	304	1.56
Chd9	chromodomain helicase DNA binding protein 9	235	367	1.56
Whrn	whirlin	194	303	1.56
Prkg2	protein kinase, cGMP-dependent, type II	442	689	1.56
Pdlim7	PDZ and LIM domain 7	399	620	1.56
Gm20806	predicted gene, 20806	121	188	1.56
Spry2	sprouty homolog 2 (Drosophila)	144	224	1.56
Hspb9	heat shock protein, alpha-crystallin-related, B9	69	107	1.55
Mrps2	mitochondrial ribosomal protein S2	220	340	1.55
Gm21333	predicted gene, 21333	205	317	1.55
Crygf	crystallin, gamma F	96	149	1.55
Itfg1	integrin alpha FG-GAP repeat containing 1	379	585	1.54
Tubb6	tubulin, beta 6 class V	378	583	1.54
Errfi1	ERBB receptor feedback inhibitor 1	636	979	1.54
Myc	myelocytomatosis oncogene	310	477	1.54
Gm21800	predicted gene, 21800 [Source:MGI Symbol;Acc:MGI:5433964]	78	120	1.54
Gm10203	predicted gene 10203 [Source:MGI Symbol;Acc:MGI:3642365]	152	234	1.54
Arhgap19	Rho GTPase activating protein 19	97	149	1.54
Ier3	immediate early response 3	416	639	1.54
Ptprj	protein tyrosine phosphatase, receptor type, J	216	331	1.53
Gm3763	predicted gene 3763	72	111	1.53

Table S3 continued.

Gene Symbol	Gene Name	Mean Intensities Ctrl	Load	Fold Change
Pde4b	phosphodiesterase 4B, cAMP specific	109	167	1.53
Il11	interleukin 11	102	156	1.53
St3gal1	ST3 beta-galactoside alpha-2,3-sialyltransferase 1	283	431	1.52
Tefm	transcription elongation factor, mitochondrial	86	131	1.52
Rragb	Ras-related GTP binding B	80	121	1.52
Gm5152	predicted gene 5152 [Source:MGI Symbol;Acc:MGI:3643407]	149	227	1.52
Klf5	Kruppel-like factor 5	122	185	1.52
Dmrtc2	doublesex and mab-3 related transcription factor like family C2	110	167	1.51
Srxn1	sulfiredoxin 1 homolog (S. cerevisiae)	298	450	1.51
Leap2	liver-expressed antimicrobial peptide 2	133	200	1.51
Gm3468	predicted gene 3468 [Source:MGI Symbol;Acc:MGI:3781644]	98	148	1.50
Fam71b	family with sequence similarity 71, member B	93	140	1.50
Ccdc51	coiled-coil domain containing 51	108	161	1.50
Crhbp	corticotropin releasing hormone binding protein	84	125	1.50
Down-regulated				
Zfp934	zinc finger protein 934	162	71	-2.30
Smo	smoothened, frizzled class receptor	396	177	-2.24
Ext2	exostoses (multiple) 2	520	262	-1.98
Ppp1r8	protein phosphatase 1, regulatory (inhibitor) subunit 8	228	121	-1.88
Zim1	zinc finger, imprinted 1	737	423	-1.74
Eepd1	endonuclease	146	84	-1.74
Cyp2d12	cytochrome P450, family 2, subfamily d, polypeptide 12	128	75	-1.70
Tmem47	transmembrane protein 47	457	273	-1.67
Erbb2ip	Erbb2 interacting protein	573	344	-1.67
Oard1	O-acyl-ADP-ribose deacylase 1	172	103	-1.66
Ddhd1	DDHD domain containing 1	261	158	-1.65
Gm14325	predicted gene 14325	701	427	-1.64
4930544G11Rik	RIKEN cDNA 4930544G11 gene	97	59	-1.64
St8sia1	ST8 alpha-N-acetyl-neuraminide alpha-2,8-sialyltransferase 1	281	174	-1.62
Stard9	START domain containing 9	780	482	-1.62
Cirbp	cold inducible RNA binding protein	217	135	-1.60
Hpgd	hydroxyprostaglandin dehydrogenase 15 (NAD)	631	396	-1.60
Krt24	keratin 24	99	62	-1.59
Lrrc41	leucine rich repeat containing 41	381	242	-1.58
F830001A07Rik	RIKEN cDNA F830001A07 gene	151	96	-1.57
Adora1	adenosine A1 receptor	197	126	-1.56
Chmp4b	charged multivesicular body protein 4B	738	472	-1.56
Ccr7	chemokine (C-C motif) receptor 7	194	125	-1.56
Abhd17b	abhydrolase domain containing 17B	144	93	-1.55
Lrrc75b	leucine rich repeat containing 75B	214	138	-1.55
Ercc5	excision repair cross-complementing rodent repair deficiency, complementation group 5	395	256	-1.54
Xylb	xylulokinase homolog (H. influenzae)	143	93	-1.53
LOC102642717	zinc finger protein 717-like	295	193	-1.53
Bmp3	bone morphogenetic protein 3	198	130	-1.53
Olfr146	olfactory receptor 146	119	78	-1.53
Pelp1	proline, glutamic acid and leucine rich protein 1	165	109	-1.52
Fam50b	family with sequence similarity 50, member B	103	68	-1.52
Fam189b	family with sequence similarity 189, member B	236	156	-1.52
Ggnbp2	gametogenetin binding protein 2	221	146	-1.51
Nrip1	nuclear receptor interacting protein 1	197	131	-1.51
Kcne2	potassium voltage-gated channel, Isk-related subfamily, gene 2	76	50	-1.51
Top2b	topoisomerase (DNA) II beta	182	121	-1.51
Adm	adrenomedullin	396	263	-1.50
Slc13a5	solute carrier family 13 (sodium-dependent citrate transporter), member 5	243	162	-1.50

Table S4 Expression of genes regulated >1.5-fold in *Ext1$^{gt/gt}$* cartilage in response to loading.

Gene Symbol	Gene Name	Mean Intensities		
		Ctrl	Load	Fold Change
Up-regulated				
Inhba	inhibin beta-A	253	1023	4.04
Gjb4	gap junction protein, beta 4	217	763	3.52
Gprc5a	G protein-coupled receptor, family C, group 5, member A	245	760	3.11
Timp1	tissue inhibitor of metalloproteinase 1	988	2518	2.55
Cd44	CD44 antigen	244	590	2.42
Ngf	nerve growth factor	336	811	2.41
Evi2a	ecotropic viral integration site 2a	106	243	2.30
Plaur	plasminogen activator, urokinase receptor	179	409	2.29
Hmga1-rs1	high mobility group AT-hook I, related sequence 1	322	701	2.18
Hbegf	heparin-binding EGF-like growth factor	334	719	2.15
Srxn1	sulfiredoxin 1 homolog (S. cerevisiae)	224	469	2.09
Dusp1	dual specificity phosphatase 1	1720	3590	2.09
Fosl1	fos-like antigen 1	255	528	2.07
Dhrs9	dehydrogenase	101	209	2.06
Sema7a	sema domain, immunoglobulin domain (Ig), and GPI membrane anchor, (semaphorin) 7A	96	199	2.06
Nt5e	5 nucleotidase, ecto	593	1214	2.05
Phlda1	pleckstrin homology-like domain, family A, member 1	310	624	2.01
Ptgs2	prostaglandin-endoperoxide synthase 2	261	494	1.90
Tnfrsf12a	tumor necrosis factor receptor superfamily, member 12a	860	1630	1.90
Ucn	urocortin	151	283	1.87
Hmga1	high mobility group AT-hook 1	253	474	1.87
Tnfaip6	tumor necrosis factor alpha induced protein 6	264	483	1.83
Ano6	anoctamin 6	234	418	1.79
Smo	smoothened, frizzled class receptor	189	336	1.78
Itga5	integrin alpha 5 (fibronectin receptor alpha)	1404	2494	1.78
Dok7	docking protein 7	178	315	1.78
Epha2	Eph receptor A2	87	154	1.78
Mat2a	methionine adenosyltransferase II, alpha	857	1482	1.73
Gm21763	predicted gene, 21763	108	187	1.73
Speer4a	spermatogenesis associated glutamate (E)-rich protein 4A	76	131	1.73
Myc	myelocytomatosis oncogene	263	452	1.72
Lurap1l	leucine rich adaptor protein 1-like	108	184	1.71
Vps45	vacuolar protein sorting 45 (yeast)	104	177	1.70
Emp1	epithelial membrane protein 1	1415	2387	1.69
Prss46	protease, serine 46	146	241	1.65
Tubb6	tubulin, beta 6 class V	336	554	1.65
Errfi1	ERBB receptor feedback inhibitor 1	597	980	1.64
Gm26605	predicted gene, 26605	81	132	1.63
Itga3	integrin alpha 3	187	304	1.63
Gm20736	predicted gene, 20736	236	383	1.62
Pkia	protein kinase inhibitor, alpha	69	111	1.61
Procr	protein C receptor, endothelial	100	161	1.61
Dusp8	dual specificity phosphatase 8	303	483	1.60
Il11	interleukin 11	89	142	1.59
Proser2	proline and serine rich 2	256	405	1.58
Qpct	glutaminyl-peptide cyclotransferase (glutaminyl cyclase)	46	73	1.58
Amy2a1	amylase 2a1	66	104	1.58
Rcan1	regulator of calcineurin 1	249	392	1.58
Prkg2	protein kinase, cGMP-dependent, type II	387	608	1.57
Igsf9b	immunoglobulin superfamily, member 9B	113	176	1.56
Sat1	spermidine	635	992	1.56
Mamld1	mastermind-like domain containing 1	263	412	1.56
Spry2	sprouty homolog 2 (Drosophila)	132	207	1.56
Exosc1	exosome component 1	124	193	1.56
Chst3	carbohydrate (chondroitin 6	272	422	1.56
Plscr5	phospholipid scramblase family, member 5	64	99	1.55
Gm7849	predicted gene 7849	106	163	1.54
Gm7861	predicted gene 7861	106	163	1.54

Table S4 continued.

Gene Symbol	Gene Name	Mean Intensities Ctrl	Load	Fold Change
Gm11236	predicted gene 11236	92	142	1.54
Jun	jun proto-oncogene	406	625	1.54
Oard1	O-acyl-ADP-ribose deacylase 1	154	237	1.54
St3gal1	ST3 beta-galactoside alpha-2,3-sialyltransferase 1	261	401	1.54
Rragb	Ras-related GTP binding B	107	165	1.54
Sfn	stratifin	155	237	1.53
Serinc4	serine incorporator 4	91	140	1.53
Pde1b	phosphodiesterase 1B, Ca2+-calmodulin dependent	407	622	1.53
Has1	hyaluronan synthase1	102	156	1.53
Htra4	HtrA serine peptidase 4	89	135	1.53
Layn	layilin	347	528	1.52
Sh3bp2	SH3-domain binding protein 2	241	367	1.52
Olfr957	olfactory receptor 957	53	81	1.51
Lce1g	late cornified envelope 1G	98	148	1.51
Schip1	schwannomin interacting protein 1	262	396	1.51
Klk10	kallikrein related-peptidase 10	174	263	1.51
Gdf5	growth differentiation factor 5	376	567	1.51
Tm4sf1	transmembrane 4 superfamily member 1	377	568	1.51
Cldn34c1	claudin 34C1	559	841	1.50
Gm3327	predicted gene 3327 [Source:MGI Symbol;Acc:MGI:3781505]	197	296	1.50
Rfx4	regulatory factor X, 4 (influences HLA class II expression)	79	119	1.50
Downregulated				
Egln3	egl-9 family hypoxia-inducible factor 3	853	358	-2.39
Gm10203	predicted gene 10203 [Source:MGI Symbol;Acc:MGI:3642365]	360	152	-2.37
Adm	adrenomedullin	527	269	-1.96
Map3k1	mitogen-activated protein kinase kinase kinase 1	439	245	-1.79
Pfkfb3	6-phosphofructo-2-kinase	479	269	-1.78
Prokr2	prokineticin receptor 2	145	83	-1.74
Hif3a	hypoxia inducible factor 3, alpha subunit	224	129	-1.74
Rnf187	ring finger protein 187	447	267	-1.67
Pla2g2e	phospholipase A2, group IIE	90	54	-1.67
Txnip	thioredoxin interacting protein	1766	1063	-1.66
Rcsd1	RCSD domain containing 1	300	181	-1.66
Igfbp5	insulin-like growth factor binding protein 5	2031	1243	-1.63
Bnip3	BCL2	1073	663	-1.62
Zfp53	zinc finger protein 53	188	116	-1.62
Idnk	idnK gluconokinase homolog (E. coli)	480	297	-1.61
Irs2	insulin receptor substrate 2	321	199	-1.61
Zfp788	zinc finger protein 788	119	74	-1.60
Nox4	NADPH oxidase 4	335	209	-1.60
Foxl1	forkhead box L1	137	86	-1.59
Agpat5	1-acylglycerol-3-phosphate O-acyltransferase 5 (lysophosphatidic acid acyltransferase, epsilon)	245	154	-1.59
Kcne3	potassium voltage-gated channel, Isk-related subfamily, gene 3	259	163	-1.59
Cd55b	CD55 molecule, decay accelerating factor for complement B	164	103	-1.59
Boc	biregional cell adhesion molecule-related	711	448	-1.59
Pacs1	phosphofurin acidic cluster sorting protein 1	253	159	-1.59
Tdrd6	tudor domain containing 6	92	58	-1.58
N4bp1	NEDD4 binding protein 1	176	112	-1.58
Lpin1	lipin 1	493	316	-1.56
Hoxa3	homeobox A3	167	107	-1.56
Bhlhe41	basic helix-loop-helix family, member e41	868	557	-1.56
Cc2d2a	coiled-coil and C2 domain containing 2A	219	141	-1.56
Pdk2	pyruvate dehydrogenase kinase, isoenzyme 2	323	208	-1.55
Zranb2	zinc finger, RAN-binding domain containing 2	302	195	-1.55
Zfp292	zinc finger protein 292	204	132	-1.54
Hykk	hydroxylysine kinase 1	130	85	-1.53
St8sia1	ST8 alpha-N-acetyl-neuraminide alpha-2,8-sialyltransferase 1	347	226	-1.53

Table S4 continued.

Gene Symbol	Gene Name	Mean Intensities Ctrl	Load	Fold Change
Cdh11	cadherin 11	351	230	-1.53
Card14	caspase recruitment domain family, member 14	134	88	-1.52
Rhobtb1	Rho-related BTB domain containing 1	122	80	-1.52
Mesp1	mesoderm posterior 1	199	131	-1.52
Metap1d	methionyl aminopeptidase type 1D (mitochondrial)	203	134	-1.51
Adhfe1	alcohol dehydrogenase, iron containing, 1	253	167	-1.51
Plekha2	pleckstrin homology domain-containing, family A (phosphoinositide binding specific) member 2	168	111	-1.51
Slfn9	schlafen 9	185	123	-1.51
Fam234a	family with sequence similarity 234, member A	303	201	-1.51
Bcl9l	B cell CLL	531	353	-1.50
Cd2	CD2 antigen	93	62	-1.50
Slc43a2	solute carrier family 43, member 2	262	174	-1.50
4930449E01Rik	RIKEN cDNA 4930449E01 gene	85	57	-1.50
Csnk1g2	casein kinase 1, gamma 2	238	159	-1.50
Pfn4	profilin family, member 4	119	79	-1.50
